# Comparison of *Mycobacterium tuberculosis* drug susceptibility using solid and liquid culture in Nigeria

**DOI:** 10.1186/1756-0500-6-215

**Published:** 2013-05-30

**Authors:** Lovett Lawson, Nanmdi Emenyonu, Saddiq T Abdurrahman, Juliana O Lawson, Gertrude N Uzoewulu, Olumide M Sogaolu, Juliana N Ebisike, Christopher M Parry, Mohammed A Yassin, Luis E Cuevas

**Affiliations:** 1 Zankli Medical Centre, Plot 1021 Shehu YarAdua Way, Abuja, Nigeria; 2 National Tuberculosis and Leprosy Control Programme, Abuja, Nigeria; 3 Nnamdi Azikiwe Teaching Hospital, Nnewi, Nigeria; 4 University College Hospital (UCH), Ibadan, Nigeria; 5 Wuse General Hospital, Abuja, Nigeria; 6 Mahidol-Oxford Tropical Medicine Research Unit, Angkor Hospital for Children, Siem Reap, Cambodia; 7 School of Tropical Medicine, Liverpool, UK

**Keywords:** Multi-drug resistant tuberculosis, First-line anti-TB drugs, Solid culture, Liquid culture, Drug sensitivity

## Abstract

**Background:**

This study compares *Mycobacterium tuberculosis* culture isolation and drug sensitivity testing (DST) using solid (LJ) and liquid (BACTEC-MGIT-960) media in Nigeria.

**Methods:**

This was a cross sectional survey of adults attending reference centres in Abuja, Ibadan and Nnewi with a new diagnosis of pulmonary tuberculosis (TB) or having failed the first-line TB treatment. Patients were requested to provide three sputum specimens for smear-microscopy and culture on LJ and BACTEC-MGIT-960. Positive cultures underwent DST for streptomycin, isoniazid, rifampicin and ethambutol.

**Results:**

527 specimens were cultured. 428 (81%) were positive with BACTEC-MGIT-960, 59 (11%) negative, 36 (7%) contaminated and 4 (1%) had non-tuberculosis mycobacteria (NTM). 411 (78%) LJ cultures were positive, 89 (17%) negative, 22 (4%) contaminated and 5 (1%) had NTM. The mean (SD) detection time was 11 (6) and 30 (11) days for BACTEC-MGIT-960 and LJ. DST patterns were compared in the 389 concordant positive BACTEC-MGIT-960 and LJ cultures. Rifampicin and isoniazid DST patterns were similar. Streptomycin resistance was detected more frequently with LJ than BACTEC-MGIT-960 and ethambutol resistance was detected more frequently with BACTEC-MGIT-960 than LJ, but differences were not statistically significant. MDR-TB was detected in 27 cases by LJ and 25 by BACTEC-MGIT-960 and using both methods detected 29 cases.

**Conclusions:**

There was a substantial degree of agreement between the two methods. However using the two in tandem increased the number of culture-positive patients and those with MDR-TB. The choice of culture method should depend on local availability, cost and test performance characteristics.

## Background

Culture of *Mycobacterium tuberculosis* complex (MtbC) is the accepted reference standard for confirmation of TB infection and is necessary for drug susceptibility testing (DST). There are several methods for culturing MtbC using solid and liquid media. Although solid media has been used for over 100 years, liquid culture media is increasingly being introduced in low and middle income countries (LMIC). Solid culture media is cheaper and more widely available, but is labour-intensive, less sensitive and slower than liquid culture [1-3]. Liquid culture systems can be automated, facilitating the processing of large numbers of specimens, but are costly and more prone to contamination [1]. The decision for a laboratory to switch from solid to liquid culture therefore should be considered carefully.

Nigeria, the most populous country in Africa, has one of the largest burdens of TB. Clinical response to treatment is often less than optimal and WHO estimates that 2.2% and 9.4% of new and re-treatment TB cases have Multi drug resistant TB (MDR-TB), ranking 15^th^ among the high MDR-TB burden countries. The Nigerian TB and Leprosy Control Programme (NTBLCP) has given high priority to improve the identification and treatment of cases with MDR-TB and has recently commissioned the development of diagnostic and treatment facilities in referral centers across the country. DST capacity however remains limited because most of these facilities are concentrated in large urban centers.

Despite the increasing availability of DST facilities in West Africa, there are very few head-to-head comparisons of the MtbC DST patterns obtained by liquid and solid systems, to inform laboratories considering whether to replace one method for the other or to run the two in parallel.

Here we compared the DST results obtained when specimens were cultured using solid (Lowenstein Jensen, LJ) and an automated liquid culture media (BACTEC Mycobacterium Growth Indicator Tube (MGIT) 960).

## Methods

This cross sectional study was conducted in Abuja, Ibadan and Nnewi, in Nigeria. The aim was to compare the isolation rate of MtbC from sputum using LJ and the BACTEC-MGIT-960 and then compare the level of agreement of DST to first-line anti-TB drugs using each type of media. Adults (>18 years old) with sputum smear-positive microscopy were enrolled between August 2009 and July 2010. Both newly diagnosed patients and those who had failed first-line treatment (patients continuing to be smear-positive after 5 months of treatment) were prospectively enrolled until 500 new and 100 re-treatment patients were enrolled. Patients enrolled were not receiving treatment at the time of enrolment. Exclusion criteria included patients unable to produce sputum or to provide informed consent to participate and smear-negative cases. The sample sizes were calculated to assess the prevalence of drug resistance in new and retreatment patients, as described in Lawson et al. [4]. Specimens considered of poor quality (salivary) or to have a quantity insufficient for both cultures were excluded.

Three sputum samples were submitted from each patient for direct Ziehl Neelsen smear microscopy. If the patient was smear-positive, the best quality specimen (defined as the most muco-purulent) was sent for culture and DST at Zankli Medical Research Laboratory (ZMRL) in Abuja within seven days of collection. Samples were refrigerated prior to shipment and maintained in cold-chain during shipment with ice packs. At ZMRL, specimens were decontaminated using Petroff’s method (4% NAOH) and cultured on LJ and on an automated BACTEC-MGIT-960 system (Beckton Dickinson, Franklin Lakes, NJ) [5,6]. Two hundred microlitres of decontaminated sputa were inoculated on each of two glycerol enriched LJ slopes and 500 μl in BACTEC-MGIT-960 7H9 bottles. The BACTEC 960 was enriched with supplement (OADC) and antibiotics (PANTA) prior to inoculation. Cultures were incubated at 37°C for up to 8 weeks on LJ and 42 days in BACTEC-MGIT-960. LJ cultures were checked weekly and BACTEC-MGIT-960 cultures were monitored continuously through the automated system. Isolates were confirmed as MtbC by ZN smear of isolates to observe the serpentine cords typical of MtbC, the Nitrate reduction test and temperature lability of the catalase test. Unfortunately these were the only available tests in the laboratory at the time of the study, as confirmatory tests such as MPT 64 BD ID test strips were not available. The time to MtbC detection on LJ and BACTEC-MGIT-960 were recorded. MGIT time to detection was defined as the interval between inoculation and the bottle being flagged as positive by the machine. LJ time to detection was defined as the time between inoculation and the culture being considered positive by naked eye reading.

Pure AFB cultures were tested for their sensitivity to streptomycin (STR), isoniazid (INH), rifampicin (RIF) and ethambutol (EMB) using the proportion method in LJ [7,8] and in MGIT bottles in the BACTEC-MGIT-960 according to the manufacturers recommendations [5,6]. Drug concentrations in LJ and BACTEC-MGIT-960 were 8.0 and 1.0 μg/ml for STR, 0.2 and 0.1 μg/ml for INH, 40 and 1.0 μg/ml for RIF and 2 and 1.0 μg/ml for EMB. The agreement of the DST results obtained using both methods were compared using Kappa statistics.

Zankli Medical Research Laboratory undergoes external quality assurance of DST for first-line anti-TB drugs through the WHO Supranational Reference Laboratory Network (SRLN) for BACTEC-MGIT-960 and LJ. The most recent EQA of the laboratory results was 100% for STR, INH, RIF and EMB when using BACTEC-MGIT-960; 100% for STR, RIF and EMB and 80% for INH when using LJ.

All data were analyzed using Epi-info (CDC, 1600 Clifton road, 1 Atlanta, GA, 30333). Proportions were compared using Chi squared tests and the degree of agreement was compared using the Kappa statistic. Ethical approval was obtained from the Federal Capital Territory Health and Research Ethics Committee and Zankli Ethical Research Review Board. Participants provided their written informed consent to participate, following the procedures approved by the ethics committees.

## Results

A total of 527 sputum specimens were cultured on LJ and the BACTEC-MGIT-960. The mean (SD, range) age of the patients was 34 (12, 7–82) years, with 315 (60%) being male. The mean (range) time to detection was 11 (1–33) and 30 (7–56) days for BACTEC-MGIT-960 and LJ, respectively. The results stratified by the participating centres are shown in Table 1. In total 527 specimens were cultured. Of these, 428 (81%) were positive with BACTEC-MGIT-960, 59 (11%) negative, 36 (7%) contaminated and 4 (1%) had non-tuberculosis mycobacteria (NTM). In comparison, 411 (78%) LJ cultures were positive, 89 (17%) negative, 22 (4%) contaminated and 5 (1%) had NTM. The mean (SD) detection time was 11 (6) and 30 (11) days for BACTEC-MGIT-960 and LJ.

**Table 1:** Culture results from BACTEC960 and LJ media by study site

	**Abuja**		**Ibadan**		**Nnewi**		**All**	
	**BACTEC960**	**LJ**	**BACTEC960**	**LJ**	**BACTEC960**	**LJ**	**BACTEC960**	**LJ**
**Positive**	160 (80%)	156 (78%)	85 (94%)	79 (88%)	183 (77%)	176 (74%)	428 (81%)	411 (78%)
**Negative**	23 (12%)	35 (17%)	2 (2%)	7 (8%)	34 (14%)	47 (20%)	59 (11%)	89 (17%)*
**Non tuberculous Mycobacteria**	2 (1%)	2 (1%)	0 (0)	0 (0)	2 (1%)	3 (1%)	4 (1%)	5 (1%)
**Contaminated**	15 (7%)	7 (4%)	3 (3%)	4 (4%)	18 (8%)	11 (5%)	36 (7%)	22 (4%)**
**Total**	200	200	90	90	237	237	527	527

* Comparison of BACTEC-MGIT-960 and LJ, p < 0.01; ** p = 0.079.

For the purpose of the head to head comparison, only patients examined with results for both culture systems were included in Table 2. The LJ and BACTEC-MGIT-960 culture results were concordant in 449 (85%) samples, with substantial agreement between the methods (Kappa = 0.7, Standard error = 0.046). Agreement included both cultures being positive in 389, negative in 49, contaminated in seven and growing NTM in four. Discordant results included nine LJ positive/BACTEC-MGIT-960 negative, 24 LJ negative/BACTEC-MGIT-960 positive and 44 contaminated by one but not the other method. The use of both methods allowed the identification of 58 cases that were positive by one method but negative by the other method. The largest proportion of additional cases (24, 41%) was LJ culture negative and BACTEC-MGIT-960 culture positive. Similarly, although 58 cultures were contaminated (22 LJ and 36 BACTEC-MGIT-960), only 7 of these were contaminated by both methods. Using both methods therefore resulted in an additional yield of cultures with interpretable results.

**Table 2:** Culture results of sputum cultured on both LJ and BACTEC-MGIT-960 system (only patients with both culture results are included)

	**LJ**			
**Bactec**	**Positive**	**Negative**	**Contaminated**	**NTM**
**Positive**	389	24	14	0
**Negative**	9	49	1	0
**Contaminated**	11	17	7	1
**NTM**	0	0	0	4

**Figure 1:**
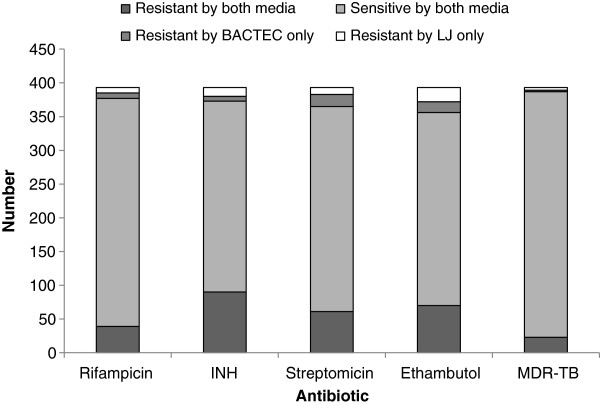
DST agreement among patients with concordant positive BACTEC-MGIT-960 and LJ cultures.

DST was conducted on both BACTEC-MGIT-960 and LJ media in the 389 concordant culture-positive isolates. The DST results obtained by LJ and BACTEC-MGIT-960 are shown in Figure 1. There were discordant results for 16 (4.1%) RIF, 20 (5.1%) INH, 28 (7.1%) STR and 37 (9.4%) EMB results. The level of DST agreement however was high and RIF and INH had equal numbers of discordant LJ and BACTEC results by each method (Kappa, 0.801 and 0.866, respectively). Streptomycin resistance was detected more frequently with LJ than BACTEC-MGIT-960 (79 versus 71 cases, respectively, Kappa 0.769) and EMB resistance was detected more frequently with BACTEC-MGIT-960 than LJ (91 and 86 cases, respectively, Kappa 0.730), but these differences were not statistically significant.

Twenty nine patients were classified as having MDR by at least one of the two methods. Of these 27 were identified by LJ and 25 by BACTEC-MGIT-960 (Kappa 0.876).

## Discussion

Tuberculosis drug resistance is increasing in many LMICs and Nigeria is not an exception [4,9,10]. Most National TB control programmes (NTP) are strengthening their capacity for the treatment of MDR-TB, but despite these steps many patients are unconfirmed because relatively few centres are equipped or have the resources to conduct DST [11].

NTPs often need to decide on the culture method that should be implemented in the country. Among the aspects that need to be considered are the sensitivity of the method and its susceptibility to contamination. The higher sensitivity of liquid over solid culture is widely accepted [12] and accordingly, in this study BACTEC-MGIT-960 was more often positive than LJ. Similarly, BACTEC-MGIT-960 often has higher contamination rates than LJ; a problem often reported from LMICs [1,2,12-14], and we also had a higher frequency of contamination in liquid culture.

Services should also be aware of the delay of both culture methods which affect clinical management. Although this delay is more prolonged with LJ, it is still significant with liquid culture and clinical decisions are often made before culture and DST results are available for both methods [15].

Liquid culture has been reported to detect more cases of first-line drug resistance than LJ by some [16] but not all studies, while others report a higher recovery rate of MTbC but similar sensitivity to LJ to detect drug resistance [3,17]. In conformity with the latter studies, we obtained a higher sensitivity of liquid culture but a high agreement between the two DST methods. The agreement was lower for STR and EMB than for RIF and INH. Although these could be due to methodological errors, our laboratory participates in an international external quality assurance system and it is likely discordant results reflect discordances of the methods, and not staff mistakes, as observed in other settings. The issue of DST discordance was the topic of a recent WHO expert group meeting, where discussion centered on whether the appropriate breakpoints were being used for some of the drugs (http://www.who.int/tb/challenges/xdr/xdrconsultation/en/index.html). If the results of both methods were considered valid, these discrepancies would result in the identification of a higher number of drug resistant strains with important cost and patient care implications. This is thus an area that requires further research.

Another consideration for choosing solid or liquid culture is the cost of the systems and the availability of consumables. Liquid culture is several times more expensive than solid media and although consumables for LJ media are universally available in Nigeria, there is only one supplier for BACTEC-MGIT-960 and we have experienced difficulties maintaining stocks. The test performance characteristics, its overall cost and availability therefore have important implications for deciding the DST method of choice in a country. In our context, and in other settings with limited resources, laboratories should be encouraged to develop solid culture capability and to add liquid culture only when the resources required become available.

The study reported here has several limitations. We only included patients with smear-positive disease and excluded smear-negative cases. This was based on the algorithm for testing in Nigeria, in which patients with smear-negative results are not routinely cultured. Smear-negative but culture-positive patients however would benefit from DST and it is possible that their exclusion could lead to some bias. This bias could be due to the yield of culture (as liquid culture may be more sensitive in smear-negative patients) and the performance of DST, as patients with paucibacillary TB or milder forms of the disease could have different DST patterns than those with high bacillary loads.

## Conclusion

In conclusion, liquid culture is more sensitive than solid culture, but there was a high degree of agreement between the two systems. Their simultaneous use resulted in a higher recovery rate and detection of more drug resistance than using only one of the two methods. The mean time to detection was shorter with liquid culture and its monitoring can be automated to facilitate dealing with large number of specimens. However this method is more costly and prone to contamination [1]. Solid and liquid culture had high levels of agreement for detecting drug resistance. Given its lower risk of contamination, costs and availability, the LJ method may be preferable in LMIC settings with limited resources and most laboratories introducing liquid culture systems should maintain solid culture until the method is well established.

## Competing interests

The authors have no conflict of interest to declare. Beckton Dickinson did not participate in the analysis, interpretation or reporting of this study.

## Authors’ contributions

LL conceived the study, conducted the statistical analysis and prepared the initial protocol. NE carried out the laboratory culture and DST at ZMC and participated in the drafting of the manuscript. STA and JOL participated in the design of the study and facilitated the study implementation in Abuja. GNU, OMS, JNE identified and recruited patients in the participating hospitals, CMP provided expert mycobacteriology expertise and interpretation of the data; MAY participated in the protocol development, data analysis and drafting the manuscript and LEC supported protocol development, data analysis and interpretation and prepared the final manuscript. All authors read and approved the final manuscript.
